# Rapid palatal expansion effects on mandibular transverse dimensions in unilateral posterior crossbite patients: a three-dimensional digital imaging study

**DOI:** 10.1186/s40510-015-0114-9

**Published:** 2016-01-08

**Authors:** Alessandro Ugolini, Tiziana Doldo, Luis T. Huanca Ghislanzoni, Andrea Mapelli, Roberto Giorgetti, Chiarella Sforza

**Affiliations:** Department of Orthodontics, University of Siena, Siena, Italy; Functional Anatomy Research Center (FARC), Laboratorio di Anatomia Funzionale dell’Apparato Stomatognatico (LAFAS), Dipartimento di Scienze Biomediche per la Salute, Facoltà di Medicina e Chirurgia, Università degli Studi di Milano, Milan, Italy

**Keywords:** Palatal expansion, Mandibular arch, 3D digital models

## Abstract

**Background:**

The purpose of this controlled study was to investigate indirect effects on mandibular arch dimensions, 1 year after rapid palatal expansion (RPE) therapy.

**Methods:**

Thirty-three patients in mixed dentition (mean age 8.8 years) showing unilateral posterior crossbite and maxillary deficiency were treated with a RPE (Haas type) cemented on the first permanent molars. Treatment protocol consisted of two turns per day until slight overcorrection of the molar transverse relationship occurred. The Haas expander was kept on the teeth as a passive retainer for an average of 6 months. Study models were taken prior (T1) and 15 months on average (T2) after expansion. A control group of 15 untreated subjects with maxillary deficiency (mean age 8.3 years) was also recorded with a 12-month interval. Stone casts were digitized with a 3D scanner (3Shape, DK).

**Results:**

In the treated group, both mandibular intermolar distance (+1.9 mm) and mandibular molar angulation (+9°) increased. Mandibular incisor angulation showed an increase of 1.9°. There was little effect on intercanine distance and canine angulation. Controls showed a reduction in transverse arch dimension and a decrease in molar and canine angulation values.

**Conclusions:**

RPE protocol has indirect widening effects on the mandibular incisors and first molars.

## Background

Posterior crossbite is one of the most prevalent malocclusions in the primary and early mixed dentition, and it is reported to occur in 8 to 22 % of the cases [1, 2]. It occurs when the maxillary back teeth bite inside the mandibular back teeth. Posterior crossbite may develop or improve at any time from when the deciduous teeth come into the mouth to when the permanent teeth come through. If the crossbite affects one side of the mouth only, the mandible may need to move asymmetrically to allow the posterior teeth to meet together. This movement may have long-term effects on the growth of the teeth and jaws. The subsequent neuromuscular adaptation to the acquired mandibular position can cause asymmetric mandibular growth, facial disharmony, and several functional changes in the masticatory muscles and temporomandibular joint (TMJ) [3]. It is unclear what causes posterior crossbites, but they may be due to skeletal, soft tissue, dental, or respiratory factors or develop as the result of a habit, e.g., thumb-sucking or some pathology. For this reason, several treatments have been recommended to correct posterior crossbite.

McNamara has speculated that the position of the mandibular dentition might be influenced more by maxillary skeletal morphology than by the size and shape of the mandible [4]. This hypothesis could explain why some mandibular arch decompensation happened during rapid maxillary expansion therapy, but very few published researches support this thesis [5–10]. While some recent investigations reviewed the palatal expansion and its effects on the palatal vault and the lower third of the face in a three-dimensional perspective, an evaluation of the effects on the mandible with a 3D noninvasive analysis is still missing [11, 12].

The primary focus of the current study was the assessment of the spontaneous mandibular response after rapid palatal expansion (RPE) therapy, in patients with unilateral crossbite, as measured from three-dimensional digital dental models.

## Methods

### Subjects

Forty-eight patients with posterior crossbite were consecutively selected. The patients were treated at the Department of Orthodontics, University of Siena (Italy) and were selected according to the following criteria:

- Early or mid mixed dentition stage
- Cervical vertebral stage 1 through 3 (CVS methods 1–3) [13]
- Unilateral posterior crossbite
- Angle Class I or Class II malocclusion
- Underwent RPE banded (Haas type) therapy (RPE, treated group) or to be submitted to RPE banded (Haas type) therapy (control group)
- No subsequent comprehensive orthodontic treatment implemented in either the maxilla or the mandible

The exclusion criteria for selection were as follows:

- Angle Class III malocclusion
- Previous orthodontic treatment
- Hypodontia in any quadrant excluding third molars
- Hormonal imbalances
- TMJ signs and/or symptoms
- Craniofacial abnormalities (e.g., cleft lip and palate)
- Arthritis

The RPE group consisted of 18 girls and 15 boys; average age at T1 was 8.8 years (sd 1.1 years). The control group consisted of 8 girls and 7 boys; average age at T1 was 8.3 (sd 1.2 years). These patients were matched for age, sex, and skeletal maturity with the RPE groups but did not receive any orthodontic treatment, and their dental casts were taken a second time after approximately 12 months.

In the RPE group, the records included pre-treatment (T1, immediately before the cementation of the appliance) and post-treatment dental casts (T2, after the appliance was removed and replaced by a removal plate, 15 months interval on average).

All palatal expanders (tooth-tissue supported, Haas type) were manufactured, cemented, and activated according to the following protocol: at initial activation, the appliances received two quarter turns (0.4 mm). Thereafter, the appliance was activated one quarter turn in the morning and one quarter turn in the evening. The subjects were seen at weekly intervals for approximately 3 weeks. When the desired overcorrection for each patient was achieved, the appliance was stabilized. Expansion was considered adequate when the occlusal aspect of the maxillary lingual cusp of upper first molars contacted the occlusal aspect of the facial cusp of the mandibular lower first molars. The expander was in situ during the expansion and stabilization period for a mean time of 7 months (range 5–9 months). After the removal of the expander, a loose, removable acrylic plate was placed within 48 h. Generally, each patient wore the acrylic plate for a variable amount of time (minimum 8 h/day).

### Cast analysis

The sample consisted of 96 cast models which were scanned by a 3SHAPE D640 SCANNER (3Shape, Copenhagen, DK) 3D digital model (*.stl) were thus obtained.

**Fig. 1:**
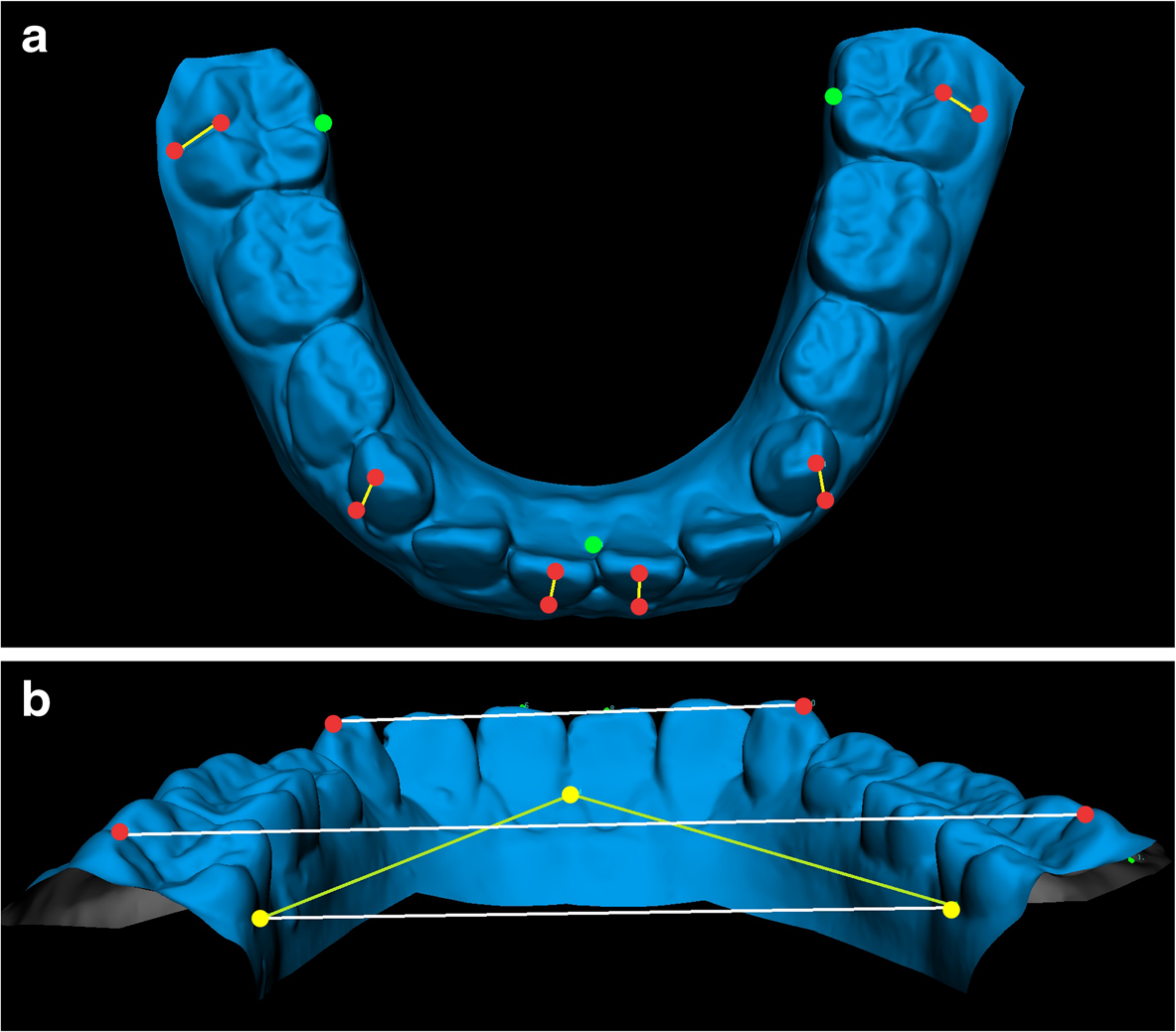
**a**, **b**. Digital model of the mandible with markers: dental markers in *red*, reference plane markers in *green*. **a** FACC, used to calculate angulation, in *yellow*. **b** Intercanine and intermolar (lingual and vestibular) distances in *white*

3D digital model processing and cast analysis were accomplished with a multi-step procedure. The first step consisted of landmark digitization on each model through VAM application version 2.8.3 (Canfield Scientific Inc., Fairfield-NJ, USA). The protocol developed by Huanca Ghislanzoni et al. [14] was followed. Dental landmarks were taken on screen on the scanned mandibular dental casts by the principal investigator (A.U.). When either the deciduous teeth were missing or the permanent teeth were not fully erupted, the measurements for that variable were eliminated. For each patient, a total of 15 mandibular landmarks were digitized (two landmarks each for the first molars, canines, and central incisors; plus 3 landmarks as reference plane). Two landmarks per teeth allowed to trace the facial axis of the clinical crown (FACC) of the first permanent molars, deciduous canines, and permanent central incisors, at T1 and at T2, respectively. Mandibular reference planes were computed between the incisive papilla and the intersections of lingual sulci of the first permanent molars with the gingival margin (Fig. 1a, b). Lingual measurements for mandibular intermolar width were obtained at the point of the intersection of the lingual groove with the cervical gingival margin, according to McDougall et al. [15] The occlusal intermolar width was measured as the distance between the mesiobuccal cusp tips of the first permanent molars bilaterally; the intercanine width was the distance between cusp tips bilaterally. Mandibular first molar, canine, and incisor angulations were calculated as the angle of projection of the FACC on the reference plane.

The whole set of landmarks was exported into a .txt file. The .txt file was imported into an Excel matrix, and x, y, and z coordinates were divided into three columns.

The 3D point set was re-orientated putting the reference lingual plane parallel to the X plane. Finally, the data set was analyzed with a custom excel procedure for 3D arch analysis. The process was repeated for each mandibular arch cast (Fig. 1a, b).

### Method error

Intraclass correlation coefficients were calculated to compare within-subjects variability to between-subjects variability; all values were larger than 0.93. Standard deviations between repeated measurements were found to be in the range of 0.08 to 0.17 mm for linear measurements and in the range of 0.5° to 1.9° for angular measurements. Overall, the method error was considered negligible.

### Statistical analysis

Descriptive statistics were computed for all analyzed variables: occlusal and lingual intermolar distances; intercanine distance; left and right molar, canine, and central incisor angulation values; and molar, canine, and incisors mean (right and left average angulation values).

Shapiro-Wilk’s test showed that data were normally distributed, and parametric statistics were applied. Patient (RPE group) data were compared with the data collected from the untreated group using Student’s *t* tests. Probabilities of less than 0.05 were accepted as significant in all statistical analyses. Sample size was calculated a priori to obtain a statistical power of the study greater than 0.85 at an alpha of 0.05, using the mean values and standard deviations of mandibular molar expansion after RPE therapy found by Lima et al. [7].

The effects size (ES) coefficient was also calculated [16]. The ES coefficient is the ratio of the difference between the recordings of two different groups (within the same recording condition) or two recording conditions (within the same group) divided by the within-subject standard deviation (sd), and it was calculated as follows:

$$ \mathrm{E}\mathrm{S}=\frac{m_{\mathrm{a}}-{m}_{\mathrm{b}}}{\surd \left[\left(\mathrm{s}{{\mathrm{d}}_{\mathrm{a}}}^2\mathrm{x}\kern0.5em {n}_{\mathrm{a}}+\mathrm{s}{{\mathrm{d}}_{\mathrm{b}}}^2\kern0.5em \mathrm{x}\kern0.5em {n}_{\mathrm{b}}\right)/\mathrm{s}{\mathrm{d}}_{\mathrm{a}}+\mathrm{s}{\mathrm{d}}_{\mathrm{b}}\right]} $$

where, *m*_a_ and *m*_b_ are the means for the generic group⁄recording conditions A and B; sd_a_ and sd_b_ are the corresponding standard deviations; *n*_a_ and *n*_b_ are the corresponding sample sizes. For Cohen’s *d*, an effect size of 0.2 to 0.3 might be a “small” effect; around 0.5, a “medium” effect; and 0.8 to infinity, a “large” effect.

A linear regression model was employed to assess correlations between treatment duration (months of therapy, MOT) and mandibular dental angulation values.

## Results

**Fig. 2:**
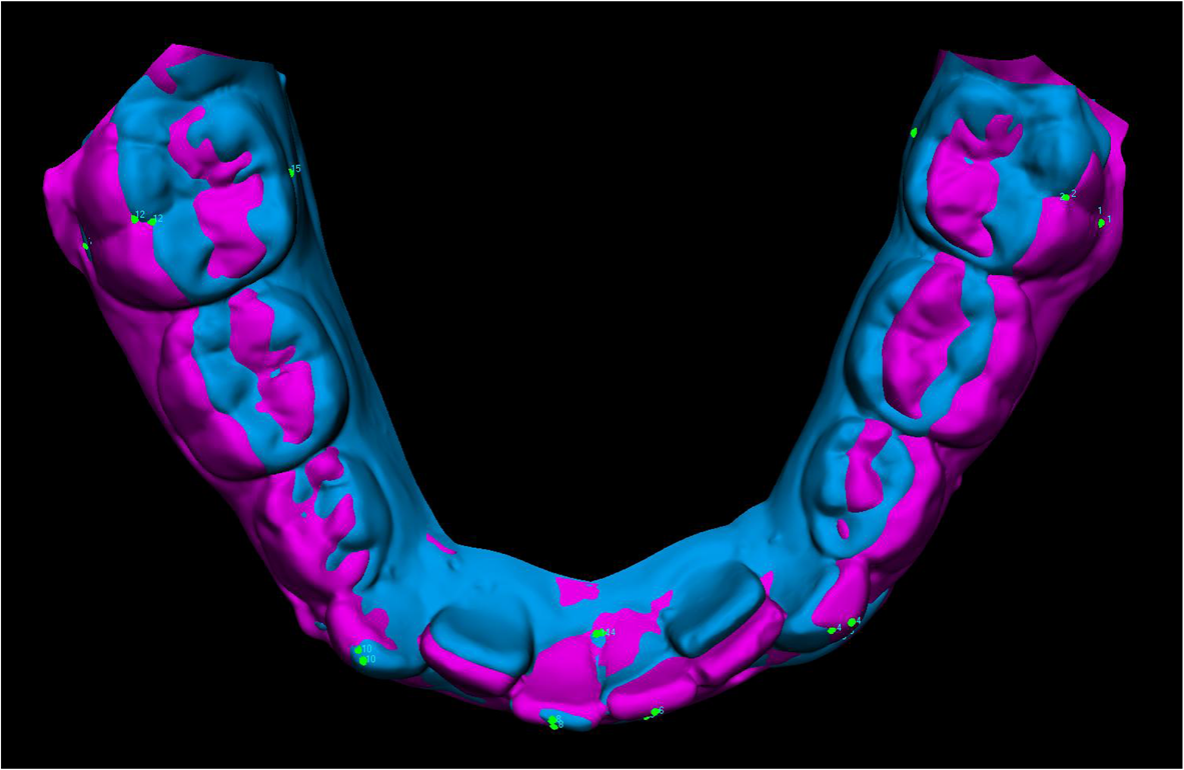
Superimposition of pre- and post-treatment digital models of the mandible shown as example of mandibular response to RPE treatment

Descriptive analyses of the mandibular variables at two assessment stages for all 48 subjects are shown in Tables 1 and 2 and Fig. 2. It was possible to measure only fully erupted teeth (permanent or deciduous). Therefore, for some measurements, a reduced number of subjects were analyzed (Table 1). No differences between groups were found at T1. At T2, all patients had their crossbite corrected. No self-crossbite corrections were observed in the control group.

**Table 1:** Descriptive statistics and comparisons between groups at T1

		Control group			RPE group		
		*n* = 15 (7 M; 8 F)			*n* = 33 (15 M; 18 F)		
Variable	Unit	*N*	Mean	sd	*N*	Mean	sd
Age	years	15	8.3	1.2	33	8.8	1.1
T1-T2	months	15	12	2.4	33	15	2.4
36–46 (occlusal)	mm	15	46.9	2.4	33	47.1	2.9
36–46 (lingual)	mm	15	33.7	1.7	33	33.5	2.4
33–43	mm	14	27.0	1.5	16	26.5	2.0
36 angulation	°	15	−44.7	6.8	33	−47.6	8.8
46 angulation	°	15	−44.7	10.7	33	−48.4	6.9
33 angulation	°	13	−13.7	6.8	20	−15.8	6.8
43 angulation	°	13	−16.3	8.9	20	−17.1	12.0
31 angulation	°	15	−8.1	4.8	25	−9.0	6.2
41 angulation	°	15	−7.7	5.4	25	−8.7	7.6

All comparisons were not significant (*p* > 0.05, Student’s *t* test for independent samples)

**Table 2:** Mean and standard deviation (sd) of the differences between T2 and T1 values for each patient

		Control group		RPE group		Diff T2-T1	*t* test	Effect size	
	Unit	Mean	sd	Mean	sd		*p* value	*d* value	ES
36–46 (occlusal)	mm	−0.8	0.8	1.1	1.5	1.9	0.00	0.6	Large
36–46 (lingual)	mm	−0.1	0.4	0.6	1.2	0.7	0.00	0.8	Large
33–43	mm	−0.6	0.8	0.4	1.6	1.0	0.01	0.4	Medium
36 angulation	°	−3.3	5.2	6.2	5.8	9.5	0.00		
33 angulation	°	−6.0	5.0	0.7	5.5	6.7	0.00		
43 angulation	°	−2.7	6.6	0.7	7.4	3.4	ns		
46 angulation	°	−3.8	5.7	4.3	6.8	8.1	0.00		
31 angulation	°	−2.5	4.0	2.0	4.1	4.4	0.00		
41 angulation	°	−2.4	3.5	1.8	3.1	4.2	0.00		
Molar angulation (mean)	°	−3.5	5.5	5.2	6.3	8.8	0.00	0.6	Large
Canine angulation (mean)	°	−4.4	5.8	0.7	6.4	5.1	0.01	0.4	Medium
Incisor angulation (mean)	°	−2.4	3.7	1.9	3.6	4.3	0.00	0.5	Medium

*Diff. T2-T1* mean differences between RPE and control groups; *ns* not significant, *p* > 0.05

*d* Cohen’s effect size value, *ES* effect size

The net changes of the T1-T2 interval are reported in Table 2. In treated (RPE group) subjects, mandibular intermolar distance significantly increased 1.9 mm on the vestibular side and 0.7 mm on the lingual side. Mandibular molar angulation increased 9°. There was a significant but little effect on mandibular incisor angulation (+1.9°), intercanine distance (+1.0 mm), and on canine angulation (+5.1°). Control subjects showed a tendency towards contraction of the transverse dimensions and a decrease in molar, canine, and inferior incisor angulation values.

ES coefficients were also calculated and are listed in Table 2. These variables (36–46 occlusal, 36–46 lingual, 33–43, molar angulation, canine angulation, incisor angulation) were characterized by a significant, medium or large, effect size.

Linear regression between MOT and mandibular first molar angulation showed a significant correlation (*p* = 0.02; *y* = 0.529*x* − 2.050, *R*^2^ = 0.441), while no correlations between MOT and mandibular central incisor and canine angulations were found.

## Discussion

All subjects were selected before the pubertal peak (CVS 1–3), because Baccetti et al. showed that in these three stages, RPE patients exhibit significant and more effective long-term changes at the skeletal level in both maxillary and circummaxillary structures [13, 17]. A control group of untreated patients with the same malocclusion was also used to identify confounding factors such as natural craniofacial growth and development during the study period.

A few data were found in biomedical literature about the RPE effects on mandibular molar, canine, and incisor angulation [10]. Otherwise, no data about changes in mandibular arch angulation in untreated unilateral crossbite malocclusion were reported in previous studies. In the current investigation, normal transversal arch growth was modified by crossbite malocclusion: the patients showed a tendency towards contraction of the transverse mandibular dimension and a decrease in molar, canine, and incisor angulation values. Previous longitudinal investigations found a slight but continued decrease in the intercanine width (0.5–1.5 mm) during the maturation of the permanent dentition [18–20]. Moorrees and Reed showed the intercanine width does not change from the age of 8 to 10 years, and the mandibular intermolar width increases 3–4 mm from 6 to 17 years of age [21].

Two long-term retrospective trials, by Geran et al. and O’Grady et al., reported the changes in untreated (Class I or Class II malocclusion but not crossbite) control groups [8, 9]. They found a reduction in mandibular arch perimeter, mainly related to the exfoliation of the mandibular second deciduous molars; a slight decrease in intercanine width and a very little or no increase in molar width. Unfortunately, the time interval (T1-T2) for decrements reported by Geran et al. for their control group was 5 years, and it cannot be directly compared to our time interval [8].

The current data allow to extend the information about longitudinal modifications in mandibular teeth angulation in untreated crossbite subjects. We found that the decrease in intercanine and intermolar width and part of arch perimeter reduction were mainly caused by the decrease in mandibular teeth angulation value.

When compared to the untreated group, the present RPE group showed significant net increases of intermolar width from pre-expansion (T1) to follow-up (T2): 1.9 mm, occlusal value, and 0.7 mm, lingual value. These increases were greater than some of the mandibular intermolar widths (occlusal) previously reported. Several authors reported an increase in mandibular molar width ranging from 0.24 to 2.8 mm [5, 6, 22, 23]. Wertz evaluated 48 patients for mandibular intermolar width changes after 3–4 months of RPE therapy (plus stabilization) and found 35 patients of 48 with no change, 12 of 48 with increases of 0.5 to 2.0 mm, and 1 of 48 with a decrease of 1.0 mm, but that study included children, teenagers, and adults [22]. Moussa et al. and Sandstrom et al. evaluated mandibular intermolar width change after RPE, but their patients also underwent fixed appliance therapy, and they are not directly comparable to our study [5, 23].

From T1 to T2, both the abovementioned increases suggest a slight first molar uprighting. This hypothesis is confirmed by the angulation values. From T1 to T2, the inferior first molar angulation was significantly increased, +8.8°. In a recent study, Lima et al., found that mandibular intermolar arch width increased significantly after RPE with a Haas-type expansion appliance and that the increase was followed by a slight decrease of the occlusal value, whereas the lingual value was maintained, thus suggesting a tendency to lingual angulation in the long term. [7]. For intercanine width (occlusal value), we found a little effect on intercanine distance (+1.0 mm) but not on canine angulation. Similar results were reported by Lima et al. [7]. Haas reported no change for intercanine width in 5 of 10 analyzed subjects; however, the age range was significant higher than in the present study [24]. All short-term and long-term studies, as reviewed by Lima et al., showed very different value for intercanine width increases, ranging from 0.5 to 5 mm, which might be attributed to differences in sample selection criteria [7]. Lagravere et al. reported that most of the mandibular intermolar increments noted immediately after RPE were not statistically significant [25].

Baysal et al. evaluated the post RPE changes in mandibular arch widths and buccolingual inclinations of mandibular posterior teeth by using cone beam computed tomography (CBCT) images. They measured linear and angular changes in mandibular posterior region and after 6 months found an increase of the axial inclinations of all mandibular posterior teeth and of the mandibular transversal dimension [10]. There is a good accord between the current and the study by Baysal et al., and data are directly comparable, due to the similar 3D measurement. Although the radiation dose of a CBCT scan is lower than that of a CT scan, CBCT is not considered suitable for all orthodontic growing patients, and it is questionable whether it is appropriate to perform more than one CBCT scan per year. Thanks to our 3D cast analysis system, we can record the same variables using noninvasive procedures.

In the present study, RPE therapy allowed an increment in mandibular arch transversal dimensions and an increase in molar, canine, and incisor angulations. Angulation increase may result from two different biomechanical effects, postulated by Haas [24]. The first is an occlusal change. The direction of occlusal forces is altered by the maxillary expansion, so that the resultant force vector acting on the mandibular teeth (especially molars) is more vestibularly directed, because the occlusal aspect of the lingual cusp of upper first molars contacts the occlusal aspect of the facial cusp of the lower first molars. The second is a “lip bumper effect”: the lateral movement of the maxillae widened the area of attachment of the buccal musculature [10]. These theses were indirectly supported by the correlation between molar angulation increase and months of therapy. Instead, the lack of correlation between MOT and incisor angulation could be related to a different tongue postural control in some patients (a possible swallowing disorder).

Although long-term longitudinal data are needed, the present study’s sample size, along with the significant effect size of the difference in the decompensation of mandibular arch, enforces the statistical significance of the outcomes.

## Conclusions

Mandibular intermolar arch width increased significantly in early mixed dentition patients with unilateral posterior crossbite after RPE (Haas-type) therapy. This increase was followed by a significant increase of molar angulation. There was a significant but little effect on intercanine distance and on canine and incisor angulations. The outcomes in spontaneous mandibular arch response to RPE showed a remarkable and positive clinical effect in mandibular arch-width dimensions in patients treated only with RPE. The molar angulation value increase was also correlated with the months of RPE therapy.

The RPE protocol has widening indirect effects on the mandibular first molars, canines, and incisors, 15 months after RPE therapy. The values of Cohen’s of effect size confirmed the clinical indirect effects of RPE on mandibular arch in early mixed dentition patients with unilateral crossbite.
